# Association of plasma potassium with mortality and end-stage kidney disease in patients with chronic kidney disease under nephrologist care - The NephroTest study

**DOI:** 10.1186/s12882-017-0710-7

**Published:** 2017-09-12

**Authors:** Sandra Wagner, Marie Metzger, Martin Flamant, Pascal Houillier, Jean-Philippe Haymann, François Vrtovsnik, Eric Thervet, Jean-Jacques Boffa, Ziad A. Massy, Bénédicte Stengel, Patrick Rossignol, for the NephroTest Study group, Emmanuel Letavernier, Pierre Ronco, Hafedh Fessi, Eric Daugas, Caroline du Halgouet, Renaud de La Faille, Christian d’Auzac, Gerard Maruani, Marion Vallet, Laurence Nicolet-Barousse, Mélanie Roland, Christian Jacquot

**Affiliations:** 1 CESP, Inserm U1018, Univ Paris-Saclay, Univ Paris-Sud, UVSQ, Villejuif, France; 2 FCRIN INI-CRCT, Paris, France; 3 Bichat Hospital, Paris, France; 4 INSERM U1138, Paris, France; 5 HEGP, Paris, France; 6 Tenon Hospital, Paris, France; 7 INSERM UMRS970, Boulogne-Billancourt, France; 8 Ambroise Paré Hospital, Boulogne-Billancourt, France; 9 INSERM CIC 1433, Nancy CHRU and University of Lorraine, Nancy, France

**Keywords:** Plasma potassium, Hypokalemia, Hyperkalemia, Chronic kidney disease, End-stage kidney disease, Mortality, Cardiovascular mortality

## Abstract

**Background:**

Low and high blood potassium levels are common and were both associated with poor outcomes in patients with chronic kidney disease (CKD). Whether such relationships may be altered in CKD patients receiving optimized nephrologist care is unknown.

**Methods:**

NephroTest is a hospital-based prospective cohort study that enrolled 2078 nondialysis patients (mean age: 59 ± 15 years, 66% men) in CKD stages 1 to 5 who underwent repeated extensive renal tests including plasma potassium (P_K_) and glomerular filtration rate (GFR) measured (mGFR) by ^51^Cr-EDTA renal clearance. Test reports included a reminder of recommended targets for each abnormal value to guide treatment adjustment. Main outcomes were cardiovascular (CV) and all-cause mortality before end-stage kidney disease (ESKD), and ESKD.

**Results:**

At baseline, median mGFR was 38.4 mL/min/1.73m^2^; prevalence of low P_K_ (<4 mmol/L) was 26.5%, and of high P_K_ (>5 mmol/L) 6.4%; 74.4% of patients used angiotensin converting enzyme inhibitors (ACEi) or angiotensin receptor blockers (ARB). After excluding 137 patients with baseline GFR < 10 mL/min/1.73m^2^ or lost to follow-up, 459 ESKD events and 236 deaths before ESKD (83 CV deaths) occurred during a median follow-up of 5 years. Compared to patients with P_K_ within [4, 5] mmol/L at baseline, those with low P_K_ had hazard ratios (HRs) [95% CI] for all-cause and CV mortality before ESKD, and for ESKD of 0.82 [0.58–1.16], 1.01 [0.52–1.95], and 1.14 [0.89–1.47], respectively, with corresponding figures for those with high P_K_ of 0.79 [0.48–1.32], 1.5 [0.69–3.3], and 0.92 [0.70–1.21]. Considering time-varying P_K_ did not materially change these findings, except for the HR of ESKD associated with high P_K_, 1.39 [1.09–1.78]. Among 1190 patients with at least two visits, P_K_ had normalized at the second visit in 39.9 and 54.1% respectively of those with baseline low and high P_K_. Among those with low P_K_ that normalized, ARB or ACEi use increased between the visits (68.3% vs 81.8%, *P* < .0001), and among those with high P_K_ that normalized, potassium-binding resin and bicarbonate use increased (13.0% vs 37.0%, *P* < .001, and 4.4% vs 17.4%, *P* = 0.01, respectively) without decreased ACEi or ARB use.

**Conclusion:**

In these patients under nephrology care, neither low nor high P_K_ was associated with excess mortality.

**Electronic supplementary material:**

The online version of this article (10.1186/s12882-017-0710-7) contains supplementary material, which is available to authorized users.

## Background

The mainstays of nonspecific secondary prevention of chronic kidney disease (CKD) progression, irrespective of cause, include blood pressure control and proteinuria-directed strategies to preserve residual kidney function, with special emphasis on angiotensin-converting enzyme inhibitors (ACEi) or angiotensin-receptor blockers (ARB) [1–4]. However, fear of inducing hyperkalemia, an inherent risk associated with the mechanism of action of these drugs, may limit their initiation or dose increases, given the considerable attention paid to this risk, especially in patients with CKD, diabetes mellitus, and or heart failure (HF) [5–8]. Although the exact serum (S_K_) or plasma potassium (P_K_) concentration associated with increased mortality remains controversial, growing evidence suggests that in patients with CKD, diabetes mellitus, or HF, especially the elderly, a S_K_ > 5.0 mmol/L is associated with a higher risk of death [9, 10]. Moreover, a *post-hoc* analysis of the Reduction of Endpoints in non-insulin-dependent diabetes mellitus with the Angiotensin II Antagonist Losartan (RENAAL) trial showed that increased S_K_ concentrations ≥5.0 mmol/L at 6 months were associated with an increased risk of doubled serum creatinine or end-stage kidney disease (ESKD), independent of baseline renal function and other important predictors of renal outcomes [11].

Low S_K_ < 4 mmol/L has also been associated with excess mortality and hospitalization, especially for patients with CKD and HF [12], for whom the relation between S_K_ and mortality is U-shaped [13]. The frequent concomitant use of non-potassium-sparing (thiazide and loop) diuretics may induce low S_K_ in CKD patients, and again a U-shaped relation has been observed between S_K_ and mortality, with mortality risk significantly greater at S_K_ < 4.0 mmol/L than at 4.0 to 5.5 mmol/L. In this CKD cohort, only the composite of cardiovascular events or death as an outcome was associated with elevated S_K_ (>5.5) [14]. Risk for ESKD was also elevated at S_K_ < 4 mmol/L. Hayes et al. reported a significant nonlinear association between S_K_ and all-cause mortality in a retrospective CKD survey; regression splines showed that mortality increased in association with both high and low S_K_ levels [15]. Other studies in CKD patients have also shown low S_K_ (<3.5 mmol/L) is associated with excess mortality [4] and ESKD risk [16]. Another study found low S_K_ (<4 mmol/L) associated with mortality in patients with CKD but not with ESKD [17]. Higher S_K_ (>5 mmol/L) was associated with excess ESKD in one study [16] but not another [17]. Nevertheless, it appears that high S_K_ (>5, 5.6 or 6 mmol/L) is associated with excess mortality [4, 17]. Of note, all these studies reported to have measured S_K_ which is known to overestimate potassium concentration on average by 0.4 mmol/L as compared with plasma potassium (P_K_) which reduces the risk for blood coagulation [18, 19].

In this study, we aimed to evaluate the association of P_K_ with renal and cardiovascular outcomes, along with treatment practice patterns in the use of drugs apt to modulate P_K_ in a cohort of patients with CKD under optimized nephrologist care, characterized by repeated extensive laboratory work-ups.

## Population and methods

### Study population

NephroTest is a prospective hospital-based cohort study that enrolled 2084 adult patients with any diagnosis of CKD stages 1–5 referred by nephrologists to three departments of physiology for extensive work-ups between January 2000 and December 2012 [20]. The NephroTest work-up was designed to optimize CKD care by providing nephrologists with a large set of blood and urine tests to assess each patient’s metabolic complications and cardiovascular risk at yearly intervals. Laboratory report notified any relevant abnormal values, such as P_K_ lower than 3.5 or higher than 5.0 mmol/L, together with a reminder of current recommended targets, to guide treatment adjustment [20].

Eligible patients were ≥18 years of age, not pregnant, not on dialysis, and not living with a kidney transplant. After exclusion of 6 patients with missing data for P_K_ or treatment at baseline, this analysis included 2078 patients (Additional file 1: Figure S1).

### Measurements

Clinical and laboratory data were recorded during a 5-h in-person visit at enrollment and during follow-up. They included demographics, renal diagnosis, medical history, height and weight, resting blood pressure, and medications. We collected blood and urine samples to determine levels of P_K_, venous CO2, HbA1c, and albumin, as well as urinary creatinine, albumin, and potassium. P_K_ status was studied in three categories: < 4 mmol/L (low P_K_), 4–5 mmol/L (normal P_K_), and >5 mmol/L (high P_K_). Diabetes was defined as either fasting glycemia ≥7 mmol/L or HbA1c ≥6.5% or antidiabetic treatment. At each visit, GFR was measured by ^51^Cr-EDTA renal clearance. Briefly, 1.8–3.5 MBq of ^51^Cr-EDTA (GE Healthcare, Velizy, France) was injected intravenously as a single bolus. An hour was allowed for distribution of the tracer in the extracellular fluid, and then the average renal ^51^Cr-EDTA clearance was determined for five to six consecutive 30-min clearance periods. Over the study period, patients underwent a total of 5523 laboratory visits, and a median of 2 [IQR, 1–4] per patient); 1190 patients (57%) had at least two visits.

### Outcomes

The primary endpoints were ESKD, defined by dialysis start or preemptive kidney transplantation, and pre-ESKD all-cause mortality. The secondary endpoints were pre-ESKD cardiovascular (CV) mortality and all-cause death, regardless of ESKD. Events were identified either from patients’ medical records or through record linkage with the national REIN (Renal Epidemiology and Information Network) registry of treated ESKD and the national death registry. All survival data were right-censored on December 31, 2013, or to the date of last visit for patients not identified in registries. Cardiovascular causes of death included ischemic heart disease, cerebrovascular disease, HF, dysrhythmia, peripheral arterial disease, sudden death, and valvular disease. Patients were followed up through December 31, 2013. These outcomes were studied in 1941 patients after exclusion of 137 with baseline GFR < 10 ml/min/1,73m^2^ or lost to follow-up from the initial sample (Additional file 1: Figure S1).

### Statistical analyses

In the overall population, we first used analysis of variance (ANOVA), the Kruskal-Wallis test, or the chi-square test, as appropriate, to compare patients’ baseline characteristics by P_K_ status subgroup. We then used multinomial logistic regression models to estimate odds ratios (OR) and their 95% confidence intervals (95% CI) for low and high P_K_ associated with baseline characteristics, with normokalemia as the reference category.

Second, we performed Cox regression models to estimate crude and adjusted cause-specific hazard ratios (HR) and their 95% confidence intervals (95% CI) for ESKD, and pre-ESKD all-cause and CV mortality associated with P_K_ status at baseline, with normokalemia [4–5 mmol/L] as the reference category. In each of these models, the competing events were treated as censored observations [21]. Adjustment covariates were similar in all analyses: age, center, sex, ethnicity, smoking status, body mass index (BMI), diabetes, baseline mGFR, albuminemia, urinary potassium, log albumin/creatinine ratio, medication that may decrease P_K_ (nonpotassium-sparing diuretics, bicarbonate treatment, potassium-binding resins), and medication that may increase P_K_ (potassium-sparing diuretics, ACEi or ARBs, β-blockers). We tested the proportional-hazard assumption with Schoenfeld residuals against time for each covariate; because it was not satisfied for mGFR in the cause-specific Cox model with ESKD, we stratified rather than adjusted for baseline mGFR level, using six classes of mGFR (10–20, 20–30, 30–40, 40–50, 50–60, >60 mL/min per 1.73m^2^). To account for changes in P_K_ over time, we used time-dependent Cox models to estimate crude and adjusted HRs for each outcome associated with P_K_ during follow-up. In the time-dependent analysis, medications were also updated at each visit. Finally, penalized splines were used in fully adjusted time-dependent Cox models to represent the functional relation between P_K_ measurements and the risk of each outcome.

Third, we described changes in P_K_ status between the first and the second visit in the subpopulation of patients with at least two visits as well as changes in medication between the visits for patients with low or high P_K_ at baseline that normalized at the second visit. Changes were tested with McNemar’s test.

Statistical analyses were performed with SAS version 9.4 (SAS Institute, Cary, NC) and R version 3.0.2.

## Results

### Baseline characteristics

The participants’ mean age at baseline was 58.8 ± 15.2 years, and the median mGFR 38.9 (27.2–53.8) mL/min per 1.73m^2^; 21.8, 21.1, 29.3, 22.0, and 5.7% of patients were in CKD stages 2, 3a, 3b, 4, and 5, respectively. P_K_ values ranged from 2.40 to 7.30 mmol/L, with a mean of 4.26 ± 0.50 mmol/L and a median of 4.20 (3.92–4.52). ACEi or ARB were prescribed to 74.4% of patients (Table 1). The distribution of P_K_ status is shown in Additional file 2: Figure S2. The prevalence of high P_K_ (>5 mmol/L) was 8.3%, and that of low P_K_ (<4 mmol/L) 27.2% (3.9% for very low P_K_ < 3.5 mmol/L).

**Table 1:** Patient characteristics according to baseline plasma potassium concentration (mmol/L)

	Overall (2078)	Baseline plasma potassium (mmol/L)			p
		<4 (*n* = 566)	4–5 (*n* = 1340)	>5 (*n* = 172)	
Demographics					
Age (years)	58.8 ± 15.2	56.0 ± 15.6	60.2 ± 14.8	56.9 ± 15.5	<.001
Men (%)	66.3	60.7	67.9	72.7	<.001
African (%)	13.0	18.0	11.0	12.2	<.001
BMI (kg/m^2^)	26.6 ± 5.2	26.2 ± 5.4	26.8 ± 5.1	26.5 ± 5.2	0.20
Former smokers (%)	30.9	25.4	33.2	30.8	<.001
Current smokers (%)	13.7	12.4	13.4	20.3	
Clinics					
CVD history (%)	17.3	10.9	19.9	18.6	<.001
Diabetes (%)	29.9	25.6	30.9	37.2	0.01
Primary kidney disease (%)					<.001
Diabetic	10.1	5.1	11.1	18.6	
Glomerular	14.1	12.4	14.3	18.6	
Vascular	26.2	26.5	26.4	23.3	
Polycystic	5.5	9.0	4.3	3.5	
Interstitial	9.0	9.9	8.8	7.6	
Other or unknown	35.0	37.1	35.0	28.5	
Measurements					
Systolic BP (mm Hg)	136.0 ± 20.4	134.7 ± 20.7	136.2 ± 20.8	138.8 ± 21.4	0.07
Diastolic BP (mm Hg)	74.8 ± 11.6	75.5 ± 12.2	74.5 ± 11.5	76.1 ± 10.3	0.13
Mean BP (mmHg)	95.3 ± 13.3	95.3 ± 14	95.0 ± 13.0	97.0 ± 12.6	0.48
mGFR (ml/min/1.73m^2^)	38.9 (27.2–53.8)	47.9 (35.7–64.2)	37.6 (26.7–51.7)	24.7 (16.4–34.3)	<.001
Albuminemia (g/L)	39.4 ± 4.5	39.4 ± 4.2	39.4 ± 4.5	39.3 ± 5.2	0.72
Albumin/creatinine ratio	8.9 (1.6–50.9)	5.4 (1.4–31.7)	9.6 (1.5–54.7)	28.0 (6.0–98.2)	<.001
Plasma potassium (mmol/L)	4.3 ± 0.5	3.7 ± 0.2	4.4 ± 0.3	5.3 ± 0.4	–
Urinary potassium (mmol/24 h)	66.0 ± 35.1	64.1 ± 31	62 ± 26.1	62 ± 26.1	0.10
Medication use					
ACEi or ARBs (%)	74.4	65.7	76.5	86.1	<.001
β-blockers (%)	37.3	32.5	39.3	38.4	0.02
Bicarbonate (%)	3.8	1.6	4.1	7.6	<.001
Potassium-binding resins (%)	6.6	2.3	7.2	16.9	<.001
Potassium non-sparing diuretics^a^(%)	46.7	48.2	45.6	50.6	0.08
Potassium-sparing diuretics^b^ (%)	3.4	4.4	3.0	2.9	0.31

Values are %, mean ± SD or median (interquartile range)

^a^Loop or thiazide diuretics. ^b^ amiloride or anti-aldosterone diuretics

*ACEi* angiotensin-converting enzyme inhibitors, *ARBs* angiotensin II receptor blocker *BMI* body mass index, *CVD* cardiovascular disease, *BP* blood pressure, *mGFR* measured GFR

Patients with high P_K_ tended to be younger, more frequently men, with a history of cardiovascular disease, diabetes, lower mGFR, and higher albuminuria, and more frequent prescriptions for ACEi, ARB, bicarbonates, or potassium-binding resins (Table 1). Those with low P_K_ were younger, more often women, and had prescriptions for those medications less often. In multivariable analyses (Table 2), higher ORs of high P_K_ were significantly associated with diabetes, current smoking, lower mGFR, and prescriptions for P_K_-increasing medication (i.e., ACEi or ARB or potassium-sparing diuretics), and lower ORs with older age and female gender. In contrast, higher ORs of low P_K_ were significantly associated with female gender and use of potassium-lowering medication, and lower Ors with lower mGFR, CVD history, and potassium-increasing medication.

**Table 2:** Odds ratios of low or high plasma potassium associated with baseline patient characteristics – Multinomial logistic regression using patients with plasma potassium of 4–5 mmol/L as the reference group)

		Plasma potassium (mmol/L)	
		<4	>5
Age (per year)		0.99 (0.98–1.00)	0.98 (0.96–0.99)
Women vs men		1.49 (1.16–1.90)	0.47 (0.30–0.72)
Sub-Saharan vs other ethnicity		1.35 (0.99–1.83)	1.15 (0.66–1.99)
mGFR (ml/min/1.73m^2^)			
	<15	0.11 (0.05–0.23)	29.65 (10.87–80.88)
	15–30	0.26 (0.18–0.38)	13.58 (5.71–32.3)
	30–45	0.47 (0.34–0.63)	5.70 (2.42–13.45)
	45–60	0.67 (0.49–0.90)	2.70 (1.07–6.85)
	>60	1	1
BMI			
	<19	1.46 (0.83–2.59)	1.49 (0.65–3.43)
	19–25	1	1
	25–30	0.85 (0.66–1.10)	0.78 (0.51–1.20)
	>30	0.78 (0.57–1.07)	1.05 (0.65–1.73)
Smoking status			
	Never smoked	1	1
	Former smoker	0.85 (0.66–1.11)	1.02 (0.67–1.54)
	Active smoker	0.76 (0.54–1.06)	1.66 (1.03–2.67)
Mean blood pressure (per mmHg)		1.01 (1.00–1.02)	1.01 (0.99–1.02)
Cardio-vascular history		0.63 (0.46–0.88)	0.81 (0.51–1.28)
ACR (mg/mmol)			
	<3	1	1
	3–30	1.07 (0.82–1.40)	1.25 (0.76–2.08)
	>30	0.80 (0.59–1.10)	1.13 (0.67–1.90)
Diabetes		0.86 (0.66–1.13)	1.56 (1.04–2.34)
Urine potassium		0.99 (0.99–1.00)	1.01 (1.00–1.01)
Serum albumin			
	≥35	1	1
	<35	1.23 (0.87–1.74)	1.23 (0.76–1.98)
Serum potassium increasing drugs^a^		0.58 (0.44–0.78)	2.50 (1.17–5.35)
Serum potassium-lowering drugs^b^		1.70 (1.33–2.17)	1.01 (0.69–1.49)

^a^ loop or thiazide diuretic, kayexalate or bicarbonates ^b^ ACEi or ARBs or potassium-sparing diuretics

BMI, body mass index, CVD, cardiovascular disease, mGFR, measured GFR, ACR, ratio of urinary albumin to creatinine

The analyses was adjusted for center

### Association of P_K_ status with ESKD and pre-ESKD mortality

**Fig. 1:**
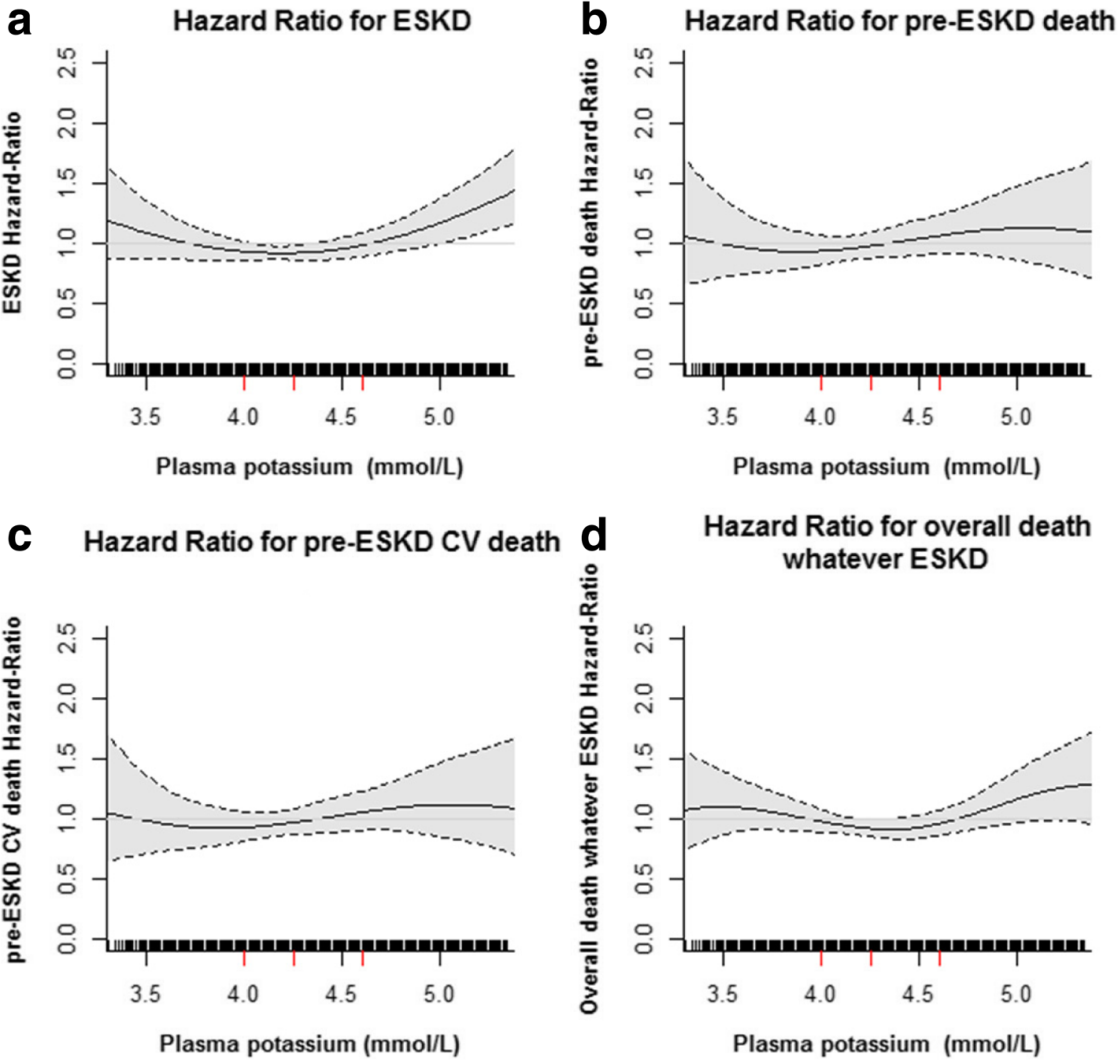
Estimated adjusted hazard ratio with 95% confidence intervals for the association of plasma potassium level with end-stage kidney disease (ESKD) (**a**), overall pre-ESKD death (**b**), and pre-ESKD cardiovascular (CV) death (**c**) and overall death whatever ESKD (**d**) using penalized-splines estimator. Hazard ratio (HR) were plotted only for values below 95th percentile

Over a median follow-up of 5 years, 459 of the 1941 patients included in this analysis began RRT for ESKD, and 236 died before starting RRT, 83 of them from CV causes. Compared to patients with normokalemia at baseline, those with low P_K_ had crude/adjusted HRs [95% CI] for ESKD and for all-cause and CV mortality before ESKD of: 0.61[0.48–0.78]/1.14 [0.89–1.47, 0.58[0.42–0.80]/0.82 [0.58–1.16], and 0.40[0.20–0.78]/1.01 [0.52–1.95], and those with high P_K_, 2.43[1.89–3.13]/0.92 [0.70–1.21], 0.97[0.59–1.60]/0.79 [0.48–1.32], and 1.41[0.67–2.97]/1.47 [0.67–3.24], respectively. The main confounder in these associations was mGFR. Considering time-varying P_K_ did not materially change these findings: adjusted HRs [95% CI] for ESKD and for all-cause and CV mortality before ESKD for those with low P_K_ were 1.14 [0.88–1.47], 0.87 [0.62–1.21], and 0.66 [0.44–1.00], and for those with high P_K_, 1.39 [1.09–1.78], 0.96 [0.59–1.57] and 0.93 [0.58–1.50], respectively. We found no significant association between P_K_ during follow-up and pre-ESKD, overall or CV mortality, or with overall mortality regardless of ESKD (Fig. 1). HRs for ESKD were slightly but significantly higher at higher P_K_ levels (>5 mmol/L).

### Changes in P_K_ status between visits

**Fig. 2:**
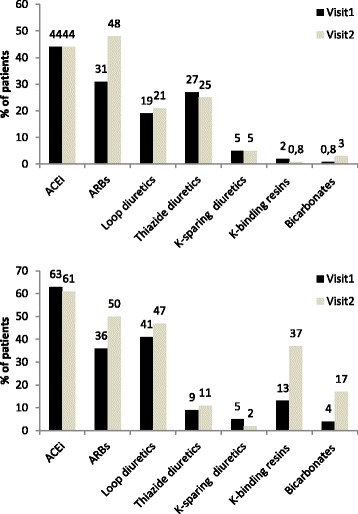
Treatment use at the first and second visit for patients having change status from low to normal plasma potassium (A - *N* = 126) and high to normal plasma potassium (B - *N* = 46). * *P*-value <0.05, ** <0.01, *** < 0.001

At the enrolment visit, 66.4% of patients were normokalemic, and at the second visit, 64.2% (Table 3). The median (q1-q3) duration between the first and the second visit was 1.26 (1.02–1.92) years. Overall, between the two visits, half of the patients remained in the normokalemic subgroup, while 39.9% of those with low P_K_ and 54.1% of those with high P_K_ at baseline had normal P_K_ at the second visit. In patients with low P_K_ that normalized, ACEi or ARB use increased between the visits (68.3% vs 81.8%, *P* < .0001) (Figure 2). In those with high P_K_ that normalized, use of potassium-binding resins and bicarbonates also rose between visits (13.0% vs 37.0%, *P* < 0.001 for potassium-binding resins, and 4.4% vs 17.4%, *P* = 0.01 for bicarbonates). The use of ACEi or ARB did not change between the two visits (80.4% at visit 1 vs 84.8% at visit 2, *P* = 0.32). Nonetheless, ARB use increased between visits 1 and 2 (36.9% vs 50.0%, *P* = 0.03).

**Table 3:** Plasma potassium level between the first and second visit

	Plasma potassium (mmol/L) at the second visit			
Plasma potassium at the first visit	<4	[4, 5]	>5	Total
<4	186 (15.6)	126 (10.6)	4 (0.3)	316 (26.6)
[4, 5]	111 (9.3)	593 (49.8)	85 (7.1)	789 (66.3)
>5	7(0.6)	46 (3.7)	32 (2.7)	85 (7.1)
Total	304 (25.6)	765 (64.3)	121 (10.2)	1190 (100%)

## Discussion

In this cohort of CKD patients under nephrologist care, low P_K_ (< 4 mmol/L) was relatively common, but hypokalemia (< 3.5 mmol/L) and high P_K_ uncommon. Neither high nor low P_K_, at baseline or during follow-up, were associated with all-cause or CV mortality in this population. A major finding from this selected cohort of patients receiving optimized nephrologist care is that the lack of excess mortality with high P_K_ was apparently observed in the absence of reduction in the use of ACEi or ARBs over time.

Optimal care of patients with CKD stage 3 or higher should involve annual assessment of metabolic and cardiovascular complications and adaptation of medication to achieve recommended therapeutic targets [22]. The NephroTest work-up implemented since 2000 in the three university hospitals in this study sought to improve CKD care by providing comprehensive assessment of CKD complications at yearly intervals together with reminders of current recommended targets. It should be emphasized that the unique design of this study with exclusive participation of patients with optimized nephrology care makes it difficult to compare our results with those from other studies. Moreover, we measured Pk which is likely to have resulted in a slight shift towards lower values as compared with other studies using Sk. A U-shaped relation has previously been reported between S_K_ and mortality in several cohorts of HF [13], hypertension [23], and CKD patients [4, 14, 15, 17], but we observed no such association with P_K_ in the NephroTest cohort.

Although no causality could be ascertained in this observational setting, we note that 74.4% of the CKD patients in our cohort were treated with ACEi or ARB at baseline (a higher rate than in the above-mentioned CKD cohorts, where it was 58.0, 59.0, and 62.1% [14, 15, 17] and 29.0% [4],), they had a low baseline prevalence of high P_K_ and a reinforced follow-up, in that patients agreed to undergo, beyond their routine nephrology care, additional extensive laboratory testing. Strikingly, low P_K_ (common at baseline) and high P_K_ (uncommon at baseline) were corrected in a substantial number of patients between the first and second NephroTest work-up: management was responsive to test results, as shown by the increased prescriptions for ARBs in patients with low baseline P_K_, and the increased prescription for potassium-binding resins and bicarbonate in those with high baseline P_K_. Interestingly, the stable ACEi and increased ARB use in these patients suggests that the nephrologists were not reluctant to prescribe drugs that might promote still higher P_K_. In contrast, a recent retrospective survey of US CKD patients reported a U-shaped association between S_K_ and discontinuation of these medications blocking the renin-angiotensin-aldosterone system (RAAS) [4]. It may be that the use of first-generation potassium-binding resins, either sodium-based (e.g., sodium polystyrene sulfonate, SPS) or calcium-based (e.g., calcium resonium), and bicarbonates made the RAAS inhibition sustainable (by taking care of the low P_K_ part of the U-shape curve) while avoiding life-threatening high P_K_ (by blunting the right-hand side of the U-shaped relation between P_K_ and outcomes). This interesting hypothesis warrants testing in randomized trials.

Only a few studies have observed a higher risk for ESKD associated with high S_K_ [11, 16]. Our study found a slight but statistically significant excess risk of ESKD at higher P_K_ levels, observed only with time-dependent Cox models. Because both P_K_ and ESKD risks rise as GFR falls, it is difficult to determine whether this reflects the potential impact of P_K_ on CKD progression or residual confounding by mGFR level.

Management of patients with chronic hyperkalemia is currently in the process of changing, and these findings are relevant to these changes [22]. Until recently, recommendations for these patients called for a low-potassium diet and the elimination of both potassium supplements and drugs, such as NSAIDS, that can compromise renal function. Instead, today, physicians are supposed to begin treatment with a non-potassium-sparing diuretic if indicated or to increase the dose for patients already on a diuretic. Dose reduction or discontinuation of RAAS inhibitors, especially mineralocorticoid receptor antagonists, is also recommended. Patients with chronic hyperkalemia for whom continued use of these drugs is thought necessary, such as those with CKD and/or HF with reduced ejection fraction, can be treated with a potassium-lowering agent such as SPS alone or with sorbitol and the RAAS-inhibitor (RAASi) treatment continued [24]. Unfortunately, the poor tolerability of available P_K_-lowering agents tends to induce poor compliance over the long run. SPS has been available to reduce potassium levels for several decades, but it is poorly tolerated and its use, especially in combination with sorbitol, has been associated with bowel necrosis [25]. Because SPS exchanges P_K_ for Na+, it can increase sodium absorption and, therefore, plasma volume, it may be dangerous in patients with volume overload such as those with chronic HF, CKD, and/or salt-sensitive hypertension. The recent availability, at least in the US, of the non-absorbed potassium-lowering polymer Patiromer and the likely availability within the year of the potassium-binding agent ZS 9 provide an opportunity to continue RAASi in patients with hypertension [25].(11) Although both Patiromer and ZS9 have been shown to be effective in reducing P_K_ to normal levels in patients with hyperkalemia and to be relatively well tolerated, their long-term effectiveness on CV and renal outcomes with continued RAASi treatment must be evaluated and compared to those outcomes in patients switching to another class of antihypertensive agent [26].

Whether the additional potassium and kidney function monitoring and reminders that were the heart of the NephroTest intervention contributed to blunting the relation between P_K_ and the outcomes tested must also be considered. Observational data certainly suggest that implementation of potassium and GFR monitoring is inadequate, even though it is recommended by all guidelines for patients treated with ACEi or ARB [27], or mineralocorticoid receptor antagonists [28].

Major strengths of our study include its large sample size and duration of follow-up, together with a high level of accuracy in patient phenotyping including the use of reference methods for measuring GFR, potassium (in plasma which is preferable to serum), and several biomarkers of metabolic complications, both at baseline and follow-up visits. Several limitations should also be noted, including its observational nature, and the percentage (6.6%) of patients excluded from the analysis because of baseline GFR < 10 mL/min/1.73m^2^ or loss to follow-up. Although this may have decreased the study power; particularly for extreme P_K_ values, it is unlikely to have biased our findings. As discussed above, the NephroTest cohort was highly selected, compared to the overall CKD patient population, a selection that precludes any generalization of our findings. Nevertheless, it was this selected nature of our population that made it possible to identify clinical practice patterns, and it is these that may lead to improved clinical management of dyskalemia in other patients. Finally, because drug doses were not recorded, we cannot document whether or not ACEi or ARB dosage was reduced when not withdrawn in patients with high P_K_.

## Conclusions

In this cohort of patients under nephrology care, low P_K_ and high P_K_ appeared to be managed dynamically over time, that is, with careful attention and responsiveness to the patient’s current metabolic status. In this context, neither low nor high P_K_ was associated with excess overall and cardiovascular mortality. Our study supports the concept perceived in clinical practice that transient abnormality in potassium levels can be controlled by appropriate interventions, and thus may not necessarily indicate the worse outcome or imply the need for discontinuation of ACE-I or ARB.

## Additional files

Additional file 1: Figure S1. Study flowchart. mGFR, measured GFR; ESKD, end-stage kidney disease. (DOCX 25 kb)

Additional file 2: Figure S2. Distribution of P_K_ levels (mmol/L) at baseline. (PNG 16 kb)

## Abbreviations

ACEi: Angiotensin converting enzyme inhibitors
ARB: Angiotensin receptor blockers
CKD: Chronic kidney disease
CVD: Cardiovascular disease
ESKD: End-stage kidney disease
HR: Hazard ratio
mGFR: measured glomerular filtration rate
OR: Odds ratio
P_K_: Plasma potassium
RAAS: Renin angiotensin aldosterone system
RAASi: RAAS inhibitor
