# Correction to: Association of plasma potassium with mortality and end-stage kidney disease in patients with chronic kidney disease under nephrologist care - The NephroTest study

**DOI:** 10.1186/s12882-017-0723-2

**Published:** 2017-10-06

**Authors:** Sandra Wagner, Marie Metzger, Martin Flamant, Pascal Houillier, Jean-Philippe Haymann, François Vrtovsnik, Eric Thervet, Jean-Jacques Boffa, Ziad A. Massy, Bénédicte Stengel, Patrick Rossignol, for the NephroTest Study group

**Affiliations:** 1 CESP, Inserm U1018, Univ Paris-Saclay, Univ Paris-Sud, UVSQ, Villejuif, France; 2 FCRIN INI-CRCT, Paris, France; 3 Bichat Hospital, Paris, France; 4 INSERM U1138, Paris, France; 5 HEGP, Paris, France; 6 Tenon Hospital, Paris, France; 7 INSERM, UMRS970, Boulogne-Billancourt, France; 8 Ambroise Paré Hospital, Boulogne-Billancourt, France; 9 INSERM CIC 1433, Nancy CHRU and University of Lorraine, Nancy, France

In the original version of this article [1], published on 12 September 2017, the explanation of ^a^ and ^b^ in the footnote of Table 2 were switched during typesetting. In this Correction Table 2, the incorrect and correct version of its footnote are shown. The affected part of the footnote is marked in italics. The original publication of this article has been corrected.

**Table 2:** Odds ratios of low or high plasma potassium associated with baseline patient characteristics – Multinomial logistic regression using patients with plasma potassium of 4–5 mmol/L as the reference group

		Plasma potassium (mmol/L)	
		<4	>5
Age (per year)		0.99 (0.98–1.00)	0.98 (0.96–0.99)
Women vs men		1.49 (1.16–1.90)	0.47 (0.30–0.72)
Sub-Saharan vs other ethnicity		1.35 (0.99–1.83)	1.15 (0.66–1.99)
mGFR (ml/min/1.73m^2^)			
	<15	0.11 (0.05–0.23)	29.65 (10.87–80.88)
	15–30	0.26 (0.18–0.38)	13.58 (5.71–32.3)
	30–45	0.47 (0.34–0.63)	5.70 (2.42–13.45)
	45–60	0.67 (0.49–0.90)	2.70 (1.07–6.85)
	>60	1	1
BMI			
	<19	1.46 (0.83–2.59)	1.49 (0.65–3.43)
	19–25	1	1
	25–30	0.85 (0.66–1.10)	0.78 (0.51–1.20)
	>30	0.78 (0.57–1.07)	1.05 (0.65–1.73)
Smoking status			
	Never smoked	1	1
	Former smoker	0.85 (0.66–1.11)	1.02 (0.67–1.54)
	Active smoker	0.76 (0.54–1.06)	1.66 (1.03–2.67)
Mean blood pressure (per mmHg)		1.01 (1.00–1.02)	1.01 (0.99–1.02)
Cardio-vascular history		0.63 (0.46–0.88)	0.81 (0.51–1.28)
ACR (mg/mmol)			
	<3	1	1
	3–30	1.07 (0.82–1.40)	1.25 (0.76–2.08)
	>30	0.80 (0.59–1.10)	1.13 (0.67–1.90)
Diabetes		0.86 (0.66–1.13)	1.56 (1.04–2.34)
Urine potassium		0.99 (0.99–1.00)	1.01 (1.00–1.01)
Serum albumin			
	≥35	1	1
	<35	1.23 (0.87–1.74)	1.23 (0.76–1.98)
Serum potassium increasing drugs^a^		0.58 (0.44–0.78)	2.50 (1.17–5.35)
Serum potassium-lowering drugs^b^		1.70 (1.33–2.17)	1.01 (0.69–1.49)

^a^ *loop or thiazide diuretic, kayexalate or bicarbonates* ^b^ *ACEi or ARBs or potassium-sparing diuretics*

BMI body mass index, CVD cardiovascular disease, mGFR measured GFR, ACR, ratio of urinary albumin to creatinine

The analyses was adjusted for center

**Table 2:** Odds ratios of low or high plasma potassium associated with baseline patient characteristics – Multinomial logistic regression using patients with plasma potassium of 4–5 mmol/L as the reference group

		Plasma potassium (mmol/L)	
		<4	>5
Age (per year)		0.99 (0.98–1.00)	0.98 (0.96–0.99)
Women vs men		1.49 (1.16–1.90)	0.47 (0.30–0.72)
Sub-Saharan vs other ethnicity		1.35 (0.99–1.83)	1.15 (0.66–1.99)
mGFR (ml/min/1.73m^2^)			
	<15	0.11 (0.05–0.23)	29.65 (10.87–80.88)
	15–30	0.26 (0.18–0.38)	13.58 (5.71–32.3)
	30–45	0.47 (0.34–0.63)	5.70 (2.42–13.45)
	45–60	0.67 (0.49–0.90)	2.70 (1.07–6.85)
	>60	1	1
BMI			
	<19	1.46 (0.83–2.59)	1.49 (0.65–3.43)
	19–25	1	1
	25–30	0.85 (0.66–1.10)	0.78 (0.51–1.20)
	>30	0.78 (0.57–1.07)	1.05 (0.65–1.73)
Smoking status			
	Never smoked	1	1
	Former smoker	0.85 (0.66–1.11)	1.02 (0.67–1.54)
	Active smoker	0.76 (0.54–1.06)	1.66 (1.03–2.67)
Mean blood pressure (per mmHg)		1.01 (1.00–1.02)	1.01 (0.99–1.02)
Cardio-vascular history		0.63 (0.46–0.88)	0.81 (0.51–1.28)
ACR (mg/mmol)			
	<3	1	1
	3–30	1.07 (0.82–1.40)	1.25 (0.76–2.08)
	>30	0.80 (0.59–1.10)	1.13 (0.67–1.90)
Diabetes		0.86 (0.66–1.13)	1.56 (1.04–2.34)
Urine potassium		0.99 (0.99–1.00)	1.01 (1.00–1.01)
Serum albumin			
	≥35	1	1
	<35	1.23 (0.87–1.74)	1.23 (0.76–1.98)
Serum potassium increasing drugs^a^		0.58 (0.44–0.78)	2.50 (1.17–5.35)
Serum potassium-lowering drugs^b^		1.70 (1.33–2.17)	1.01 (0.69–1.49)

^a^ *ACEi or ARBs or potassium-sparing diuretics* ^b^ *loop or thiazide diuretic, kayexalate or bicarbonates*

BMI, body mass index, CVD, cardiovascular disease, mGFR, measured GFR, ACR, ratio of urinary albumin to creatinine

The analyses was adjusted for center

Originally Table 2 and its footnote were published as followed:

The analyses was adjusted for center

The correct version of Table 2 and its footnote is:

The analyses was adjusted for center
