# Supplementary material for: Association of plasma potassium with mortality and end-stage kidney disease in patients with chronic kidney disease under nephrologist care - The NephroTest study
Source: BMC Nephrol. 2017 Sep 12;18:295. doi: 10.1186/s12882-017-0710-7 (PMC5596852; doi:10.1186/s12882-017-0710-7)
Supplement: Supplementary file 1 — Study flowchart. mGFR, measured GFR; ESKD, end-stage kidney disease. (DOCX 25 kb) [file 12882_2017_710_MOESM1_ESM.docx]

2078 patients

1941 patients

baseline GFR<10 mL/min/1.73m² or lost to follow-up (n=137)

2084 patients

Missing data at inclusion for

- Plasma potassium (n=4)
- Treatment (n=2)

*Associations with ESKD or death before ESKD*

*Descriptive statistics*
