# Supplementary figures and images for: Association of plasma potassium with mortality and end-stage kidney disease in patients with chronic kidney disease under nephrologist care - The NephroTest study
Source: BMC Nephrol. 2017 Sep 12;18:295. doi: 10.1186/s12882-017-0710-7 (PMC5596852; doi:10.1186/s12882-017-0710-7)

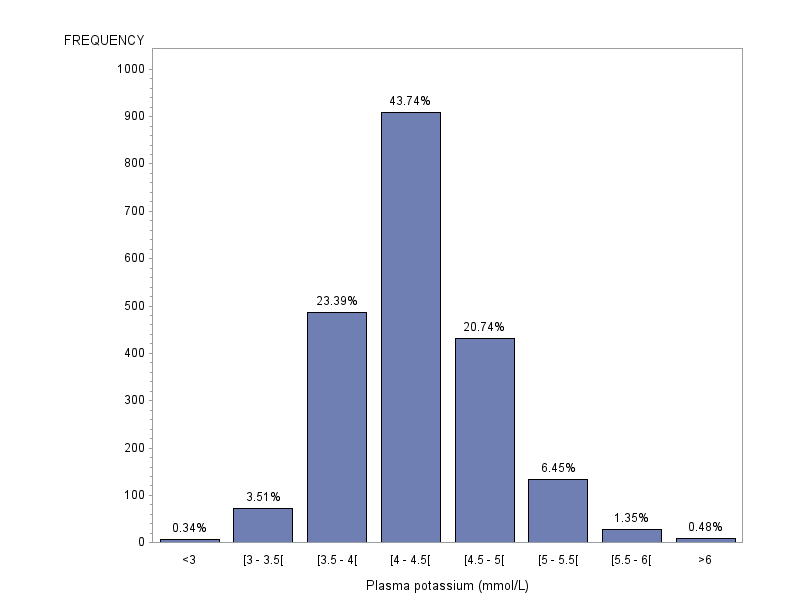

Supplement: Supplementary file 2 — Distribution of PK levels (mmol/L) at baseline. (PNG 16 kb) [file 12882_2017_710_MOESM2_ESM.png]
